# Association of the Composite Inflammatory Biomarker GlycA, with Exercise-Induced Changes in Body Habitus in Men and Women with Prediabetes

**DOI:** 10.1155/2017/5608287

**Published:** 2017-05-31

**Authors:** David B. Bartlett, Cris A. Slentz, Margery A. Connelly, Lucy W. Piner, Leslie H. Willis, Lori A. Bateman, Esther O. Granville, Connie W. Bales, Kim M. Huffman, William E. Kraus

**Affiliations:** ^1^Duke Molecular Physiology Institute, Duke University School of Medicine, Durham, NC, USA; ^2^Division of Medical Oncology, Department of Medicine, Duke University, Durham, NC, USA; ^3^LipoScience, Laboratory Corporation of America Holdings, Morrisville, NC, USA; ^4^Physical Medicine and Rehabilitation Service, Veterans Affairs Medical Center, Durham, NC, USA; ^5^Division of Cardiology Medicine, Duke University Medical Center, Durham, NC, USA

## Abstract

GlycA is a new composite measure of systemic inflammation and a predictor of many inflammatory diseases. GlycA is the nuclear magnetic resonance spectroscopy-derived signal arising from glucosamine residues on acute-phase proteins. This study aimed to evaluate how exercise-based lifestyle interventions modulate GlycA in persons at risk for type 2 diabetes. GlycA, fitness, and body habitus were measured in 169 sedentary adults (45–75 years) with prediabetes randomly assigned to one of four six-month exercise-based lifestyle interventions. Interventions included exercise prescription based on the amount (energy expenditure (kcal/kg weight/week (KKW)) and intensity (%VO_2peak_). The groups were (1) low-amount/moderate-intensity (10KKW/50%) exercise; (2) high-amount/moderate-intensity (16KKW/50%) exercise; (3) high-amount/vigorous-intensity (16KKW/75%) exercise; and (4) a Clinical Lifestyle (combined diet plus low-amount/moderate-intensity exercise) intervention. Six months of exercise training and/or diet-reduced GlycA (mean Δ: −6.8 ± 29.2 *μ*mol/L; *p* = 0.006) and increased VO_2peak_ (mean Δ: 1.98 ± 2.6 mL/kg/min; *p* < 0.001). Further, visceral (mean Δ: −21.1 ± 36.6 cm^2^) and subcutaneous fat (mean Δ: −24.3 ± 41.0 cm^2^) were reduced, while liver density (mean Δ: +2.3 ± 6.5HU) increased, all *p* < 0.001. When including individuals in all four interventions, GlycA reductions were associated with reductions in visceral adiposity (*p* < 0.03). Exercise-based lifestyle interventions reduced GlycA concentrations through mechanisms related to exercise-induced modulations of visceral adiposity. This trial is registered with Clinical Trial Registration Number NCT00962962.

## 1. Introduction

When combined with a dietary intervention, physical activity can be an effective means of slowing the progression to type 2 diabetes mellitus (T2DM) [1–3]. Combined lifestyle changes make it difficult to distinguish differences between the dietary and the exercise components. Although Pan et al. [4] suggested that exercise alone can reduce the risk of diabetes and is comparable to diet alone or diet+exercise, most interventions assess changes in risk factors, including glucose, lipids, and inflammation [5–9]. As such, even with minimal weight loss, exercise in participants who are overweight and sedentary has beneficial effects on cardiometabolic risk [5–8]. Often, responses to exercise interventions are assessed with combined measures of body composition, lipoproteins, and proinflammatory biomarkers; such markers include interleukin 6 (IL-6), tumor necrosis factor-*α* (TNF-*α*), and C-reactive protein (CRP) [10, 11]. Although the use of these inflammatory risk factors as defining measures of clinical health status is well documented, their responses to lifestyle and exercise interventions are not clear [12–18].

GlycA is a marker of systemic inflammation, measured via nuclear magnetic resonance spectroscopy (NMR), whose signal arises from the N-acetyl glucosamine residues on the glycans of acute-phase proteins (APPs) [19, 20]. Although the GlycA signal is associated with concentrations of IL-6, TNF-*α*, fibrinogen, and CRP, these proteins contribute negligibly to concentrations of GlycA [20, 21]. GlycA levels are raised in acute febrile illnesses [22]. In addition, GlycA is positively correlated with body mass index (BMI), insulin resistance, markers of metabolic syndrome, and the ratio of leptin to adiponectin, suggesting that it is related to adipose tissue-associated low-grade inflammation [23, 24]. As such, GlycA is a biomarker for cardiometabolic disease risk [25]. In support of this claim are data that show that GlycA is associated with CVD in a secondary prevention cardiovascular cohort (CATHGEN) [26] and with incident CVD events in the Women's Health Study (WHS) [27], the Prevention of Renal and Vascular End-Stage Disease (PREVEND) study [28], the Multi-Ethnic Study of Atherosclerosis (MESA) [29], and the JUPITER trial [30], independent of traditional CVD risk factors. Notably, following adjustment for traditional risk factors (e.g., BMI, LDL, and age), these associations were often only slightly attenuated by CRP, implying that GlycA measures CVD risk independently of CRP and may be a marker of a different inflammatory process. GlycA was also associated with incident T2DM [31, 32] even after adjusting for clinical T2DM risk factors. GlycA concentrations are higher in patients with inflammatory autoimmune diseases such as rheumatoid arthritis (RA) [33, 34], systemic lupus erythematosus (SLE) [35, 36], and psoriasis [37]. Moreover, GlycA is associated with disease activity [33, 34] and coronary atherosclerosis in RA [33] as well as active vascular inflammation in psoriasis [37]. Taken together, these data support the concept that GlycA is a marker of systemic inflammation and a risk factor for cardiometabolic disease. To date, no study has assessed the effects of exercise-based interventions on GlycA concentrations in an at-risk population in an attempt to understand how exercise might modulate this unique risk factor.

The purpose of Studies of Targeted Risk Reduction Interventions through Defined Exercise-Prediabetes (STRRIDE-PD) was to compare the effects of different amounts and intensities of exercise without diet to a Clinical Lifestyle (exercise plus diet) program modeled after the first six months of the Diabetes Prevention Program (DPP) [1]. The outcomes were focused on cardiometabolic risk factors in individuals at-risk for diabetes. Because little is known about the effects of diet and exercise on the GlycA biomarker, we sought to evaluate GlycA levels in the STRRIDE-PD study, determine whether exercise alone or exercise plus diet would favorably modify circulating GlycA concentrations, and try to understand how exercise effects might be mediated.

## 2. Methods

### 2.1. Study Design

Study design and experimental procedures have been described previously [9]. Briefly, 195 sedentary 45- to 75-year-old adults were recruited with a BMI between 25 and 35 kg/m^2^ and identified as having prediabetes by two consecutive fasting plasma glucose measurements between 95 and 125 mg/dL taken one week apart. GlycA measures were not available for all 195 recruits due to either insufficient plasma sample volume for NMR analysis or samples that did not produce an NMR spectrum that was good enough for GlycA quantification. Specifically, interferents can arise in the NMR spectrum that do not allow quantification of various analytes. Interferents can arise from such things as medication use or hemolysis of the sample. Therefore, of the 195 recruited into the study, only 169 participants had GlycA measured at both time points and these participants are reported here. Exclusion criteria included an inability to exercise, smoking, diabetes, uncontrolled hypertension, musculoskeletal disorders, and/or cardiovascular disease. The protocol was approved by the Duke Institutional Review Board and informed written consent was obtained.

### 2.2. Exercise and Dietary Interventions

Participants were randomized by gender and race (white women, white men, nonwhite women, and nonwhite men) into one of the four six-month intervention groups; exercise mode was predominantly using treadmills and also included elliptical trainers, rowing, and bicycle ergometers. VO_2_ reserve was chosen for exercise prescription and calculated as previously described [38]. The Clinical Lifestyle program, a combined diet and exercise program, is based on the Diabetes Prevention Program which resulted in a better risk reduction for diabetes than metformin [1]. In effect, this is the gold standard lifestyle intervention for reducing the risk of diabetes.

1. Low-amount/moderate-intensity exercise (Low-Mod)—exercise energy expenditure of 10 kcal per kg per week (KKW) at 50% VO_2_ reserve
2. High-amount/moderate-intensity exercise (High-Mod)—16 KKW at 50% VO_2_ reserve
3. High-amount/vigorous-intensity exercise (High-Vig)—16 KKW at 75% VO_2_ reserve
4. Clinical Lifestyle intervention (Clinical Lifestyle)—10 KKW at 50% VO_2_ reserve plus a calorically restricted diet designed to reduce body weight by 7% over six months.

### 2.3. Fitness and Body Composition

Fitness and body composition were evaluated at baseline and at the end of six months of prescribed exercise training. Exercise treadmill testing was used to determine cardiorespiratory fitness as previously described [39]. Briefly, aerobic capacity (VO_2peak_) was determined by a graded maximal treadmill test which started at 3 mph/0% grade and then increased speed and/or grade such that the metabolic demand increased at approximately 3.5 mL/kg/min until volitional exhaustion. Height was measured to the nearest 0.1 cm using a stadiometer (Seca-220, Hamburg, Germany). Body composition was assessed according to Siri's three-compartmental model [40]. Body weight was first assessed using a calibrated scale before assessment of body fat and lean tissue mass with air-displacement plethysmography (BodPod System; Life Measurement Corporation, Concord, CA) [41, 42]. Waist circumference was measured around the minimal waist and blood pressure was taken following 15 minutes sitting quietly.

### 2.4. Abdominal Computerized Tomography (CT)

*S*ingle 10 mm thick axial sections of the abdomen were scanned before and after the intervention using a General Electric CT/I scanner (GE Medical Systems, Milwaukee, WI). Liver density and abdominal cross-sectional areas for visceral adipose tissue (VAT) and subcutaneous adipose depots were assessed using OsiriX© Software (Pixmeo, Geneva, Switzerland). The determination of adipose tissue depots and liver density by CT are very accurate, precise, and reliable [43, 44].

### 2.5. Plasma Measures

Peripheral venous blood was drawn into vacutainers containing EDTA and placed in ice. Blood was centrifuged at 1600 ×*g* at 4°C for 10 minutes and plasma flash frozen in liquid nitrogen and stored at −80°C until analyses was conducted. Plasma samples were acquired following an overnight fast and at between 16 and 24 hours following their final exercise session. Plasma GlycA, glucose, and insulin were quantified as previously described [9, 33]. Briefly, glucose was measured by a clinical analyzer (Beckman Coulter Dx C600, Brea, CA, USA) and insulin by electrochemiluminescent plate assay (Meso Scale Discovery, Gaithersburg, MD, USA). GlycA was assessed by NMR spectroscopy, where NMR spectra were acquired from plasma samples as previously described for the NMR Lipoprofile (lipoprotein particle distribution) at LipoScience, Laboratory Corporation of America Holdings (Raleigh, NC, USA) [45]. The NMR Profiler platform is comprised of a 9.4 T (400 MHz ^1^H frequency) spectrometer (Bruker Biospin, Billerica, MA, USA) with an integrated fluidics sample delivery system. The intra-assay and interassay variability for GlycA measurement is 1.9% and 2.6%, respectively [20].

### 2.6. Statistical Analyses

All analyses were conducted using PASW v21.0 (Chicago, IL, USA) and all data are presented as mean (SD) unless otherwise stated. Normality was assessed using Kolmogorov-Smirnov analysis and natural log transformation of variables violating normality was completed. Repeated measures ANOVA were used to assess changes in outcomes. Models included group, time, and group-by-time interactions. Sex differences were assessed at baseline and for change scores with a univariate ANOVA. There were no differences for change scores between men and women and so, data was not split by sex for each group. Even though we have low power to detect significant interactions in four training groups, we assessed GlycA changes within groups with pair-wise post hoc comparisons correcting for multiple comparisons using the method of Bonferroni. Linear regression analysis of log-normalized data was used to test significant associations between changes in outcome measures and changes in GlycA. Following our analyses of GlycA, a sample size estimation was performed with change in GlycA as an outcome (GPower v3.1.9.2, Universitat Dusseldorf, Germany) [46]. Using our correlation between baseline and follow-up values of 0.804, calculated partial *η*^2^ of 0.035 which gave an effect size of 0.190, it would have taken 52 participants in each of the four groups to observe significant differences between groups. Statistical significance was accepted at *p* ≤ 0.05.

## 3. Results

We previously reported [9] metabolic and cardiorespiratory fitness indices in the whole cohort (*n* = 195). Here, we report similar findings for the 169 participants who had GlycA reliably assessed at both time points.

### 3.1. Baseline Analyses

Baseline characteristics for the entire group and by intervention are shown in Table 1. There were no differences between groups for any of the data presented (all *p* > 0.05). As expected, men and women differed on baseline cardiorespiratory fitness, body fat percentage, and subcutaneous and visceral adiposity (all *p* < 0.001), data not shown. As previously reported, when compared to men, women had greater concentrations of GlycA (*p* < 0.001), data not shown [33]. Although we have previously reported exercise prescription for the study [9], Table 2 highlights the prescription and adherence values for the 169 participants who had GlycA measured at both time points.

### 3.2. Effect of Intervention

#### 3.2.1. Cardiorespiratory Fitness

**Figure 1:**
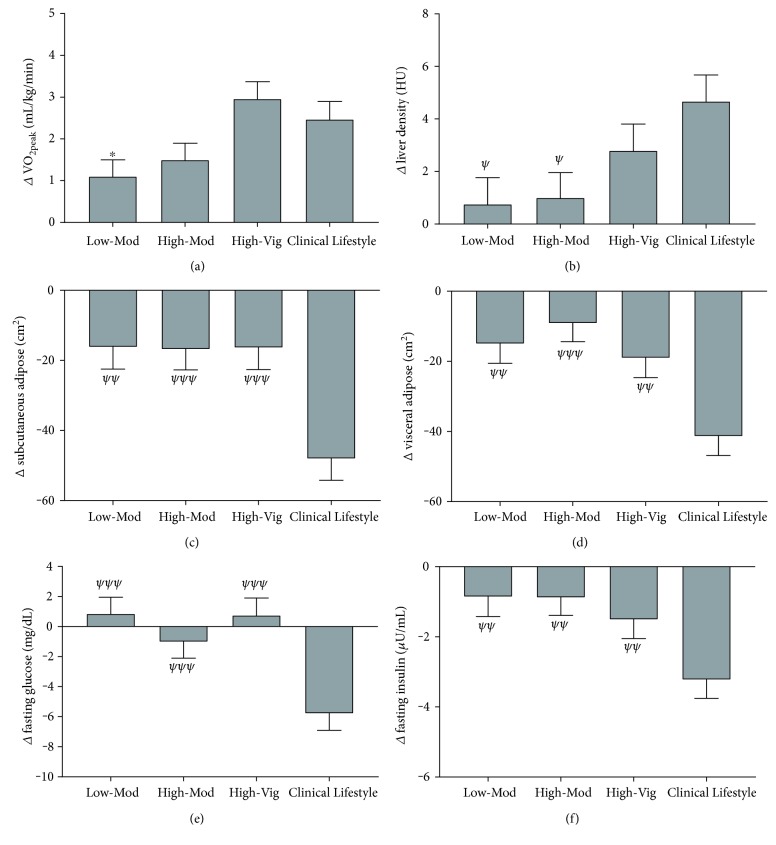
Mean (SEM) change scores for VO_2peak_ (a), liver density (b), subcutaneous (c) and visceral adiposity (d), fasting glucose (e), and insulin (f) for each intervention. ^∗∗^*p* < 0.01 different from the High-Vig group; ^ψ^*p* < 0.05; ^ψψ^*p* < 0.01; ^ψψψ^*p* < 0.001 different from Clinical Lifestyle.

As expected, there was a significant main effect for time (F(1, 168) = 95.3, *p* < 0.001, *η*^2^ = 0.390) with increases in absolute VO_2peak_ for each group following the interventions (all *p* < 0.01), Figure 1(a). There were significant group × time interactions (F(3, 165) = 4.4, *p* < 0.01, *η*^2^ = 0.082); following post hoc analyses, this was because the High-Vig group increased VO_2peak_ to a greater extent than the Low-Mod group (*p* = 0.011) and a trend towards greater than the High-Mod group (*p* = 0.08).

#### 3.2.2. Body Composition

For liver density, a surrogate measure of liver adiposity, there was a significant main effect for time (F(1, 168) = 18.6, *p* < 0.001, *η*^2^ = 0.105) with liver density increased by 5% in the High-Vig group (*p* = 0.002) and 8% in the Clinical Lifestyle group (*p* < 0.001), Figure 1(b). There were significant group × time interactions (F(3, 165) = 3.3, *p* = 0.021, *η*^2^ = 0.06); following post hoc analyses, this was because liver density increases were greater in the Clinical Lifestyle group compared to that in the High-Mod (*p* = 0.048) and Low-Mod (*p* = 0.025) but not that in the High-Vig (*p* = 0.163) group. For subcutaneous adiposity, there was a significant main effect for time (F(1, 168) = 68.6, *p* < 0.01, *η*^2^ = 0.303) with reductions observed in each group (Figure 1(c); all *p* < 0.01). For visceral adiposity, there were was a significant main effect for time (F(1, 168) = 69.0, *p* < 0.001, *η*^2^ = 0.304) with reductions observed in each group (Figure 1(d); all *p* < 0.05). There were significant group × time interactions for subcutaneous (F(3, 165) = 7.7, *p* < 0.001, *η*^2^ = 0.127) and visceral fat reductions (F(3, 165) = 7.0, *p* < 0.001, *η*^2^ = 0.118); which was because of greater reductions in subcutaneous and visceral fat in the Clinical Lifestyle intervention compared to that in every other group (all *p* < 0.01).

#### 3.2.3. Metabolic Measures

For fasting glucose, there was a significant main effect for time (F(1, 168) = 5.3, *p* = 0.022, *η*^2^ = 0.035) with glucose reduced by 5% in only the Clinical Lifestyle group (*p* < 0.001). There was a significant group × time interaction (F(3, 165) = 7.6, *p* < 0.001, *η*^2^ = 0.136) because of greater reductions in the Clinical Lifestyle group compared to that in every other group (all *p* < 0.001), indicating that diet, rather than exercise, was responsible for reduced fasting glucose, Figure 1(e). For fasting insulin, there was also a significant main effect for time (F(1, 168) = 36.4, *p* < 0.001, *η*^2^ = 0.201) with reductions in the Low-Mod (*p* = 0.015), the High-Vig (*p* = 0.005), and the Clinical Lifestyle (*p* < 0.001) groups, Figure 1(f). There was a significant group × time interaction (F(3, 165) = 4.4, *p* = 0.005, *η*^2^ = 0.083) because Clinical Lifestyle was better at reducing fasting insulin than any of the other groups (all *p* < 0.01), indicating that exercise alone can reduce insulin but exercise and diet combined are superior at reducing fasting insulin.

#### 3.2.4. GlycA

**Figure 2:**
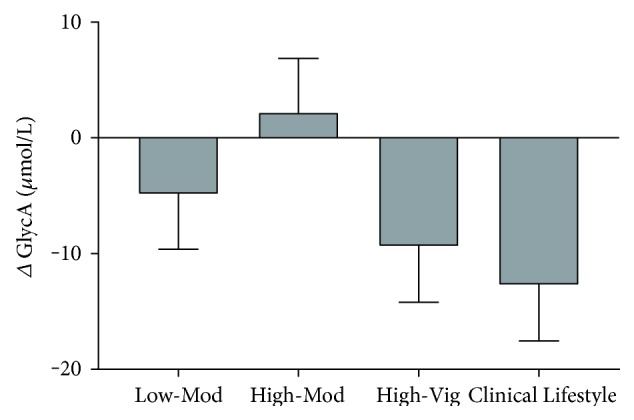
Mean (SEM) change scores for GlycA. Although there was a significant effect for time (*p* = 0.006) with an overall 2% reduction in GlycA, no differences were observed between groups (*p* > 0.05).

For GlycA, there was a significant main effect for time (F(1, 168) = 7.9, *p* = 0.006, *η*^2^ = 0.05), with an average GlycA reduction of 2% among the whole cohort, Figure 2. There were no group × time interactions and no differences between groups for absolute changes in GlycA (F(3, 165) = 2.0, *p* = 0.123, *η*^2^ = 0.035). As this study is assessing responses for a novel biomarker of chronic inflammation and is hypothesis generating, we felt obliged to perform Bonferroni-corrected pair-wise analyses on the within-groups change for GlycA. This would give some indication of how individual groups were contributing to the 2% reduction in GlycA and allows the readers a chance to draw their own conclusions. Our analyses revealed that GlycA was reduced on average by 3% in the High-Vig group (*p* = 0.033) and on average by 4% in the Clinical Lifestyle intervention (*p* = 0.007). The Low-Mod group reduced GlycA on average by 1% (*p* = 0.305) while the High-Mod group increased GlycA on average by 1% (*p* = 0.705).

#### 3.2.5. Relationships

To investigate the contributions of various physiological improvements to GlycA responses, we evaluated relations between GlycA and changes in fitness, liver density, and various measures of body composition (Table 3). Reductions in GlycA were associated with reductions in BMI (*p* = 0.03), body fat percentage (*p* = 0.03), total abdominal adiposity (*p* = 0.02), and visceral adiposity (*p* = 0.02). Furthermore, reductions in GlycA were associated with reduced fasting insulin concentrations (*p* = 0.01) but not fasting glucose (*p* = 0.23). As BMI, body fat percentage, and visceral adiposity are inter-related, we conducted multivariable analyses with changes in fasting insulin and visceral adiposity. Multivariable regression analysis indicated that changes in GlycA were somewhat associated with changes in reductions in visceral adiposity (*p* = 0.06) but not with fasting insulin (*p* = 0.124). Taken together, these data suggest an anti-inflammatory response in our interventions—effects likely mediated by reductions in ectopic fat more than by improved insulin sensitivity.

## 4. Discussion

We describe, for the first time, the effects of exercise-based lifestyle interventions on a newly described composite measure of systemic inflammation, GlycA. Further, this is associated, at least in part, with exercise-induced modifications of ectopic fat stored in the visceral compartment. We were marginally underpowered to detect differences between groups for change in GlycA. However, as this is the first study to examine responses of GlycA to exercise-based lifestyle interventions, we are compelled to present as complete a story as we can and give our best interpretation. We realize that others may offer alternative interpretations and future research should aim to determine with an adequately powered randomized control trial the influence of exercise and/or diet on GlycA. In overweight individuals with prediabetes, a six-month lifestyle intervention of either exercise alone or exercise combined with diet resulted in a small (2%) significant reduction in circulating GlycA. Without a control (no intervention) group, it is unclear from our analyses which groups were responsible for the changes or whether simply any intervention would result in a change in GlycA. Our interpretation suggests that a Clinical Lifestyle (combined exercise and diet) program and a similar period of high-amount vigorous-intensity exercise significantly contributed to reductions in plasma GlycA concentrations. However, we cannot discount that with more participants or longer periods of training, low-amount moderate-intensity or high-amount moderate-intensity exercise might also reduce GlycA concentrations. In Table 3, all intervention groups are combined to assess relations between physiologic responses to exercise-containing regimens and GlycA. These analyses imply that lifestyle interventions that modify GlycA do so primarily in relation to their ability to modify ectopic energy stores—primarily those in the visceral compartments. Although the strengths of the associations were small, with baseline GlycA the largest predictor of follow-up concentrations, when controlling for this these small associations remain significant. This would explain why adding a diet regimen to reduce body fat can augment the effects of exercise interventions on changes in systemic inflammation as measured by GlycA.

Also of interest is the observation that when matched for energy expenditure (High-Vig and High-Mod), the effects of vigorous intensity exercise outperform those of moderate intensity exercise. This establishes a contribution of exercise intensity on the responses and incorporates the near significant association between exercise-induced changes in peak VO_2_ (*p* = 0.06; Table 3). It is well established and confirmed in this study that when matched for energy expenditure, vigorous intensity exercise induced a greater increase in cardiorespiratory fitness (Figure 1).

### 4.1. Acute-Phase Protein Responses to Exercise and Diet

The GlycA signal originates from specific glycan residues found on APPs, primarily *α*1-acid glycoprotein (AGP), haptoglobin, *α*1-antitrypsin, *α*1-antichymotrypsin, and transferrin [20]. CRP, IL-6, and fibrinogen contribute negligibly to the GlycA signal, and any changes in these APPs and cytokines are unlikely to impact our findings [20]. APPs serve as regulators of inflammation through various functions such as collating iron and reducing oxidative damage, reduction of aberrant tissue damage by inhibiting proteases, and providing efficient pharmacokinetics [47–49]. Most circulating APPs are N-linked glycoproteins whose glycan structures are modified during both acute and chronic inflammatory conditions [48, 50, 51]. In support of APP glycosylation as a risk factor for disease, synthesis and glycosylation of AGP, haptoglobin, and transferrin are associated with a number of chronic diseases such as pancreatitis, rheumatoid arthritis, diabetes, and inflammatory lung conditions [52–54]. Additionally, in a large population-based study, a recently created NMR-based risk score, which includes AGP, was found to predict all-cause and CVD mortality [55]. Given that GlycA concentrations reflect a composite measure of systemic inflammation rather than concentrations of individual APPs, it may be a more consistent and accurate method to determine overall inflammatory and cardiometabolic disease risk than traditional markers [33].

To date, no studies have assessed the response of GlycA to exercise training. However, the responses of specific APPs—such as AGP and haptoglobin—to exercise training and/or diet have been studied. Prescott and colleagues showed no change in AGP following 8 weeks of exercise in elderly heart failure patients [56]. However, they also reported no change in body composition or aerobic fitness, supporting the premise that changes in body composition, particularly visceral adiposity, mediate the observed exercise-induced changes in APPs. In a recent study by Lavoie and colleagues, the combination of high physical activity levels and superior dietary quality, assessed by the Canadian Healthy Eating Index (C-HEI), was associated with reductions in the cardiometabolic risk factors CRP and apolipoprotein B in postmenopausal women [57]. This study also reported reductions in APPs known to contribute to the GlycA signal. Specifically, high physical activity levels with good C-HEI scores were associated with reduced concentrations of haptoglobin while lower AGP was associated with reduced fat mass.

Together, the studies by Lavoie, Prescott, and ours suggest that APP reductions are associated with fat loss and exercise-induced modification of cardiorespiratory fitness. In support, GlycA is associated with the leptin/adiponectin ratio [23]. The leptin/adiponectin ratio is a surrogate marker of adipose tissue function, and higher ratios are associated with a dysfunctional phenotype that contributes to inflammation and insulin resistance in nondiabetic individuals [58]. Visceral adipose tissue is a component of the metabolic syndrome and is a major producer of IL-6, TNF-*α*, and MCP-1 [59, 60]. Therefore, it is possible that our interventions reduced visceral adiposity enough to impact the secretion and enhanced glycosylation of circulating APPs.

### 4.2. GlycA Changes in Relation to Health and Disease

We report here a small but significant reduction in plasma GlycA concentration. Whether this reduction is relevant to disease risk remains unknown; however, a number of cross-sectional studies suggest that small changes in GlycA are associated with risk of disease and mortality. Most recently, hazard ratio (HR) risk of mortality from colorectal cancer was 1.24 per SD increment in GlycA [61]. Interestingly, GlycA ranges of 327–369 *μ*mol/L was associated with an HR of 0.92, 370–416 *μ*mol/L was associated with an HR of 1.26, and over 416 *μ*mol/L was associated with an HR of 1.46, suggesting that small increments in GlycA concentrations are physiologically relevant for risk of mortality [61]. Furthermore, the difference in GlycA between patients with rheumatoid arthritis, a highly inflammatory disease, and matched controls was 20–30 *μ*mol/L (353 ± 67 versus 329 ± 54) suggesting that small differences have significant disease associations [34]. GlycA was measured in the PREVEND study, and the highest GlycA quartile (>384 *μ*mol/L) was at a significantly increased risk of developing type 2 diabetes compared to the lowest quartile (<306 *μ*mol/L) [32]. Taken together, we suggest that small GlycA changes may be physiologically relevant and even small reductions may reflect reduced risk of inflammatory-mediated damage that is observed in many chronic diseases.

### 4.3. GlycA Responses and Vigorous Intensity Exercise

One observation points to the contribution of exercise intensity to the exercise-training response: the High-Vig group did not reduce visceral adiposity more than the moderate intensity groups, even when matched for energy expenditure. This may be due to the lack of adequate statistical power to detect these differences. A previous work with larger numbers of participants has shown both greater fat depot improvements with larger amounts of exercise than our study [62, 63]. Therefore, it is possible that reductions in GlycA in the High-Vig group could be influenced by exercise intensity-induced visceral adipose loss and increased cardiorespiratory fitness. In young individuals, exercise-induced APP responses are specified by exercise intensity, duration, mode, and timing of APP measurements following the last exercise exposure [64–66]. Liesen and colleagues observed exercise intensity-specific responses to an acute bout of exercise in many of the APPs contributing to GlycA [66]. Of interest, acute bouts of exercise induced smaller increases in AGP and haptoglobin following nine weeks of higher intensity endurance exercise training with no significant weight loss. These findings suggest that these APPs can be regulated by the intensity of exercise, independent of body weight changes.

### 4.4. GlycA and Hepatic Fat

The primary source of APPs is hepatic synthesis and steatosis can be accompanied by steatohepatitis. Assessing liver fat content is difficult; gold standard needle biopsies are invasive and present a risk of mortality [67]. Over the last 20 years, the use of computed tomography to determine liver fat content has become a safer and an easily accessible alternative to biopsies [68, 69]. With a good correlation between CT scans and needle biopsies [70], the lower the attenuation of the liver, the lower the tissue density and thus, the greater the fat content. As such, liver density is an established surrogate measure for liver fat content [71].

Our data suggests that liver fat was reduced in both the High-Vig and Clinical Lifestyle groups. There are clear links between liver fat accumulation and insulin sensitivity, glucose control, inflammation, and the risk of T2DM [72–74]. Therefore, as the High-Vig and Clinical Lifestyle groups reduced fasting insulin and subcutaneous and visceral fat, and as the Clinical Lifestyle also reduced fasting glucose, it would seem likely that ectopic liver fat was reduced in both groups. These findings are similar to our previous study showing, as compared to a control group of no exercise, that aerobic exercise training was associated with increased liver density (lower fat) and reduced plasma alanine aminotransferase (ALT), which is associated with reduced hepatic insulin sensitivity and risk for T2DM [75, 76]. However, a consequence of each of our four lifestyle interventions is an exercise and/or dietary modification of metabolism. With this change in metabolism comes the potential to generate confounding assumptions from CT data. Changes in water content, depending on the analyses technique and dispersion of measured molecules, could result in assumptions of either increased or decreased fat content [77, 78]. We are unaware of exercise studies assessing CT-derived liver density in response to extreme changes in hepatic water only content, such as dehydration. Acute exercise with no carbohydrate intake and dieting are associated with temporal changes in liver metabolite (i.e., glycogen) content [79, 80]. As glycogen is stored with water, these changes could potentially influence liver density. In their very elegant review paper discussing liver glycogen metabolism responses to exercise, Gonzalez and colleagues extracted data from three studies and suggest that unlike muscle glycogen content, there are no differences in basal liver glycogen content between those with T2DM, healthy controls, or endurance-trained athletes [80–82]. Together, these data suggest that it is unlikely that our interventions resulted in noticeable increased (or decreased) glycogen storage, which would confound our interpretation of fat content. Instead, glycogen utilization during exercise would be improved following a period of training, which would contribute to an increased energy reserve during exercise [79]. Therefore, although it is possible that changes in liver density are a reflection of changes in metabolite content, potentially explaining the lack of association with density and GlycA, we do not believe this to be the case. Instead, from our previous data and studies of correlations between liver density and liver adiposity, plus our observation of subcutaneous and visceral fat reductions, we believe that the majority of changes can be attributed to reduction in liver fat content. The role that liver fat and other fat depots play on glycosylation of APPs remains unclear; future work should determine the relationship of ectopic fat and GlycA in order to understand better the role of glycosylation in health and disease.

### 4.5. Limitations

We recognize this study has several limitations. We have already discussed the limitations of the GlycA analyses and the lack of a control group. Although we have speculated about the possible mechanisms whereby exercise modifies the APP components of GlycA, we are unable to determine the molecular and cellular physiology whereby exercise-based interventions actually modify GlycA concentrations. Future work should aim to determine which organs and biological processes underlie these adaptations. For instance, one might ask whether the reductions in GlycA plasma concentrations are evident because of reductions in concentrations of particular APPs or through the reduction in glycosylation of specific acute-phase proteins—or both. This will be important when designing lifestyle interventions for individuals at risk of chronic disease.

## 5. Conclusion

GlycA is a composite and accurate measure of systemic inflammation and is reduced in individuals at risk for the development of T2DM following 6 months of an exercise-based lifestyle intervention. These adaptations appear to be mediated by intervention-induced reductions in ectopic fat stores—particularly in the visceral compartment—coupled in part with favorable modifications of cardiorespiratory fitness. The implications of these studies for disease prevention are significant. Increased GlycA is associated with incidence of T2DM and CVD and with vascular disease in patients with RA and psoriasis [27, 31–33, 37]. Thus, diet and/or exercise-induced GlycA reductions may reflect a reduced risk of developing T2DM and diabetes-associated cardiometabolic complications for a number of inflammatory conditions. Which type (mode, volume, and intensity) of exercise and/or diet that modifies GlycA remains unclear and critically does a better job than another. The current study suggests that a 6-month exercise-based lifestyle intervention which improves cardiorespiratory fitness and reduces body fat content can reduce GlycA concentrations. Our study is relevant to the future of personalized prescription of exercise and diet in clinical populations who find such interventions difficult to maintain.

## Figures and Tables

**Table 1:** Baseline demographics, fitness, body composition, inflammation, glucose, and insulin for each intervention group.

	Total group (*n* = 169)	Low-Mod (*n* = 41)	High-Mod (*n* = 45)	High-Vig (*n* = 40)	Clinical Lifestyle (*n* = 43)
Age (years)	59 (7.5)	57 (7.9)	61 (6.8)	61 (7.0)	58 (7.9)
Men/women	67/102	17/24	18/27	15/25	17/26
Race					
Caucasian	132	32	36	31	33
African American	29	9	8	7	5
Others	8	0	1	2	5
Fitness					
VO_2peak_ (mL/kg/min)	24.5 (5.1)	25.0 (5.8)	24.2 (5.0)	23.9 (5.2)	24.9 (4.5)
Body composition					
Body mass index (kg/m^2^)	30.5 (2.7)	30.6 (2.6)	30.0 (2.4)	30.4 (2.7)	31.0 (3.2)
Minimal waist (cm)	98.7 (8.8)	98.3 (8.0)	98.2 (8.2)	99.8 (8.6)	98.6 (10.2)
Body fat (%)	40.8 (7.8)	39.7 (8.0)	40.9 (6.7)	41.5 (8.2)	41.0 (8.4)
Total abdominal adiposity (cm^2^)	523 (110)	517 (89)	522 (102)	523 (137)	528 (112)
Subcutaneous (cm^2^)	330 (97)	327 (81)	324 (89)	335 (127)	335 (92)
Visceral (cm^2^)	192 (73)	190 (83)	198 (68)	188 (72)	193 (71)
Liver density (HU)	59 (10)	60 (8)	60 (7)	56 (13)	59 (10)
Blood pressure					
Systolic BP (mmHg)	126 (14)	126 (14)	127 (15)	128 (13)	124 (14)
Diastolic BP (mmHg)	75 (10)	77 (9)	74 (7)	75 (11)	75 (11)
Inflammation					
GlycA (*μ*mol/L)	348 (47)	348 (51)	342 (47)	351 (44)	352 (47)
Glucose and insulin					
Fasting Glucose (mg/dL)	106 (9)	106 (11)	106 (8)	104 (8.5)	106 (10.9)
Fasting insulin (*μ*U/mL)	7.4 (5.0)	7.3 (3.6)	6.7 (4.6)	7.7 (6.7)	7.9 (4.8)

Data are mean (SD). No significant differences observed between groups.

**Table 2:** Exercise prescription and actual completed exercise for each group.

	Total group	Low-Mod	High-Mod	High-Vig	Clinical Lifestyle
Exercise prescription (Rx)					
Intensity (% peak VO_2_)		50	50	75	50
Amount (KKW)		10	16	16	10
Time (min/week)	212 (63)	179 (35)	290 (55)^***^	194 (37)	179 (35)
Exercise completed					
Adherence (% of Rx time)	85 (17)	89 (16)	82 (16)	85 (17)	85 (19)
Amount (KKW)	11 (3.2)	9 (1.9)	13 (2.6)	14 (2.7)	9 (1.9)
Frequency (sessions/week)	3.3 (0.8)	3.1 (0.7)	3.9 (0.9)^***^	3.2 (0.7)	3.0 (0.7)

KKW (kcal per kg per week). Data are mean (SD). ^∗∗∗^*p* < 0.001 different from all other groups

**Table 3:** Associations of changes in fitness, body composition, liver density, fasting glucose, and insulin on changes in GlycA concentrations.

	Change in log GlycA		
	R^2^ adj	B (95% C.I.) × 10^3^	*p*
Fitness			
VO_2peak_ (mL/kg/min)	0.627	−2.0 (−4.0, 0.0)	0.06
Body composition			
BMI (kg/m^2^)	**0.645**	**4.0 (0.0, 8.0)**	**0.03**
Body fat (%)	**0.616**	**2.0 (0.0, 3.0)**	**0.03**
Total abdominal adiposity (cm^2^)^a^	**0.642**	**120 (23.0, 217)**	**0.02**
Subcutaneous (cm^2^)^a^	0.631	88 (−11.0, 18.8)	0.08
Visceral (cm^2^)^a^	**0.637**	**79 (15.0, 14.2)**	**0.02**
Liver density (HU)^a^	0.629	−28.0 (−121, 66.0)	0.56
Glucose and insulin			
Fasting glucose (mg/dl)^a^	0.621	129 (−84, 342)	0.23
Fasting insulin (*μ*U/mL)^a^	**0.63**	**44 (11, 77)**	**0.01**

^a^Log transformed

## Abbreviations

AGP: *α*1-acid glycoprotein
ALT: Alanine aminotransferase
APP: Acute-phase-protein
C-HEI: Canadian Healthy Eating Index
CRP: C-reactive protein
CT: Computerized tomography
CVD: Cardiovascular disease
DPP: Diabetes Prevention Program
IL: Interleukin
KKW: kcal per kg per week
MCP: Monocyte chemoattractant protein
PREVEND: Prevention of Renal and Vascular End-Stage Disease
STRRIDE-PD: Studies of Targeted Risk Reduction Interventions through Defined Exercise-Prediabetes
T2DM: Type 2 diabetes mellitus
TNF: Tumor necrosis factor
WHS: Women's Health Study.
